# Use of emerging technologies in rehabilitation education and practice

**DOI:** 10.3389/fresc.2025.1655454

**Published:** 2025-08-11

**Authors:** Cristina Dumitrescu

**Affiliations:** OT Division of Decker College, Binghamton University, Binghamton, NY, United States

**Keywords:** virtual reality, digital competency, teaching tool, neuroscience education, active learning, higher-education

## Introduction

Virtual Reality (VR) is increasingly recognized as an emerging and transformative tool in neuroscience teaching in rehabilitation education and higher education in general. By offering immersive, interactive learning environments, VR enables students to explore the brain's structure-function relationships in ways that traditional instructional methods are less successful. Traditional teaching methods, including textbooks and two-dimensional diagrams, may fail to convey the complexity of neuroanatomy and its details. VR addresses these limitations by offering three-dimensional visualization, interactivity, and experiential learning, fostering critical thinking and deeper cognitive engagement.

This paper presents an opinion on the strategic integration of VR into neuroscience education. The argument is grounded in a shift from memorization-based learning to active application, analysis, and synthesis, skills increasingly emphasized in health professions training. VR supports this shift by enabling students to dynamically analyze brain-behavior relationships, engage with simulated clinical scenarios, and cultivate clinical reasoning in safe, repeatable environments. These benefits are particularly relevant as students prepare to apply neuroscience knowledge to real-world decision-making in practice settings. Despite its advantages, challenges with technology costs, accessibility, and the need for instructor training remain significant barriers to the adoption of VR in neuroscience teaching. Faculty development and pedagogical preparedness are critical to successfully implementing VR in education.

To participate meaningfully in emerging digital pedagogies, learners and educators must develop a foundational level of digital literacy. A useful framework for conceptualizing this need draws on an analogy from linguistics. While native English speakers require approximately 12,000 words for full fluency, non-native speakers often communicate effectively with just 4,000, about 30% of the total lexicon (1). This “30% rule” suggests that functional competence can be achieved through targeted, foundational knowledge rather than comprehensive mastery. Applied to digital education, this principle reinforces the idea that students do not need to become technology experts; instead, they need to achieve functional digital fluency, sufficient to interact meaningfully with tools like VR in complex learning environments (1).

Developing baseline digital literacy is increasingly essential in rehabilitation education and practice, not only for navigating instructional technologies such as virtual reality but also for engaging in evidence-based, tech-enabled care. As digital tools become embedded in both the classroom and the clinic, cultivating functional digital fluency among students and educators serves as a foundational competency, supporting professional readiness, interdisciplinary collaboration, and meaningful participation in the ongoing digital transformation of healthcare education and service delivery.

As neuroscience advances and health professions education adapts to the demands of clinical practice, the need for instructional methods that bridge theory and application becomes urgent. One promising technology is VR, which meets this need by enabling active exploration, immediate feedback, and multimodal learning. VR allows students to investigate brain structures within an immersive and interactive setting, enhancing their comprehension of neuroanatomy and neural function (2). By offering a 3D view of the brain, VR enables learners to manipulate and study neural structures from various angles, promoting a more intuitive understanding of their organization and connectivity. Immersive learning through VR boosts student engagement, encourages active exploration, and offers real-time feedback, thereby addressing multiple limitations of traditional methods (3). As educational institutions increasingly embrace technological advancements to enhance learning experiences, VR has become a vital resource in neuroscience instruction. However, despite its promise, integrating VR into neuroscience education presents challenges, including technological hurdles, accessibility concerns, and the necessity for instructor training (4). Additionally, the effectiveness of VR based instruction is contingent upon instructor competency; faculty members must be adequately trained to integrate VR into their teaching methodologies effectively. Ensuring accessibility and inclusivity is also a pressing concern, as not all students may have equal access to VR technology due to financial or physical constraints (3). Ultimately, the integration of VR into neuroscience curricula has the potential to revolutionize the way students perceive, interact with, and understand the complexities of the human brain, leading to more effective and engaging educational experiences.

### Aligning virtual reality with contemporary learning theories

The pedagogical value of VR in neuroscience education is well supported by contemporary learning theories that emphasize active, experiential, and situated engagement. As health professions education evolves to prioritize clinical reasoning, professional identity development, and real-world application of content, VR emerges as an instructional tool that directly supports these pedagogical goals. This technological approach aligns with constructivist and experiential learning theories, which emphasize learning through direct interaction rather than passive reception of information (5). This type of interaction enhances knowledge construction and supports long-term retention (4). Embodied cognition, a theory that links motor experiences and cognitive processes, offers further support for immersive learning. As students physically interact with virtual structures using motion controls and spatial navigation, they deepen their understanding of neuroanatomical configurations and functional connectivity Students can manipulate 3D brain structures, observe functional networks in action, and engage in real-time problem-solving, thus reinforcing knowledge retention and comprehension (6).

Constructivist learning theory remains central to immersive learning. Rooted in the idea that learners construct knowledge through active engagement with their environment, constructivism aligns well with the interactive nature of VR. In immersive neuroscience instruction, students manipulate virtual models of the brain, explore spatial relationships between anatomical structures, and observe simulated neuropathology. Such engagement supports deeper conceptual understanding and knowledge retention. Radianti et al. (4) emphasize that immersive environments in higher education allow learners to actively construct meaning through direct interaction, which is particularly advantageous in complex subject areas like neuroanatomy.

Closely related is the framework of experiential learning, which emphasizes learning as a cyclical process involving concrete experience, reflective observation, abstract conceptualization, and active experimentation. When applied in a VR enhanced neuroscience course, experiential learning enables students to engage with realistic representations of the nervous system, simulate clinical scenarios such as stroke or traumatic brain injury, and reflect on the implications for occupational performance. Tudor Car et al. (7) note that immersive learning experiences support critical thinking and professional skill development by replicating authentic clinical challenges in a safe, repeatable environment.

The concept of embodied cognition further supports the use of VR in occupational therapy education. Embodied cognition posits that cognitive processes are deeply influenced by the body's interactions with the environment. In VR, students physically navigate and manipulate neurological structures, linking motor actions to conceptual understanding. This embodied interaction enhances spatial reasoning and supports the formation of durable mental models. Makransky and Petersen (8) found that embodied interaction in immersive environments significantly improved learners' understanding of spatially complex information, underscoring the value of VR in anatomy-based learning.

Cognitive load theory also plays a critical role in the design of immersive learning experiences. Tudor Car et al. (7) emphasize that instructional designers must balance intrinsic, extraneous, and germane cognitive load to optimize learning outcomes. In the context of neuroscience education, this may involve sequencing learning activities from simple to complex, integrating multimodal feedback, and providing scaffolding to support novice learners. Its ability to foster cognitive engagement, promote reflection, and situate learning within authentic, embodied experiences directly addresses the instructional challenges inherent in teaching neuroscience across rehabilitation disciplines. These kinesthetic engagements activate sensory pathways that support deeper learning, an advantage not easily replicated in traditional classrooms (7).

Another pertinent framework is situated learning theory, which emphasizes that learning is most effective in context-rich, authentic environments. Immersive simulations allow students to engage in clinical decision-making scenarios that reflect real-world occupational therapy practice. For example, students may assess functional deficits associated with cortical damage or observe the effects of neuroplasticity in response to implemented treatment interventions. According to Jensen & Konradsen (3), such simulations increase learners' perception of authenticity, motivation, and engagement, thereby enhancing the transfer of knowledge to clinical practice.

These theories articulate a compelling rationale for integrating VR into occupational therapy curricula. They highlight how immersive learning environments can foster content mastery and the development of professional identity, empathy, and clinical reasoning. When grounded in theoretical frameworks, the use of immersive technologies in neuroscience education becomes a deliberate pedagogical strategy that aligns with the goals of transformative graduate-level instruction.

Beyond theoretical implications, VR offers tangible, practical benefits in neuroscience education. It provides enhanced spatial understanding, allowing learners to appreciate the three-dimensional complexities of the brain in a way that traditional illustrations cannot convey (2). Moreover, VR based simulations offer opportunities for students to apply theoretical knowledge in clinically relevant scenarios, bridging the gap between classroom learning and real-world practice (9).

Looking ahead, the future of VR in neuroscience education holds great promise. Advances in VR technology, including the development of more affordable and portable devices, will likely facilitate broader adoption. The integration of artificial intelligence within VR platforms has the potential to further personalize learning experiences, adapting to individual student needs and providing targeted feedback (10). Future research should focus on evaluating the long-term impact of VR on student learning outcomes, including retention, engagement, and skill acquisition. Additionally, interdisciplinary collaboration among educators, neuroscientists, and technologists will be essential in refining VR applications to align with evolving pedagogical needs (11).

### Immersive learning and VR

As immersive learning becomes increasingly integrated into health profession education, aligning it with clear, measurable learning outcomes is essential. To measure learning outcomes effectively in immersive environments, assessment strategies must be adapted to capture both performance and reflection. Traditional assessments such as multiple-choice exams may not fully capture the depth of learning occurring in VR based activities. Instead, performance-based assessments, practical exams, structured reflections, and rubrics aligned with clinical reasoning frameworks are more appropriate. According to Tudor Car et al. (7), combining immersive learning with formative assessments, such as self-paced simulations with real-time feedback, can significantly improve learner outcomes in health professions education.

Embedded assessments within VR environments are also gaining traction. These assessments capture learner interactions, choices, and progression in real time. For example, a neuroscience VR module may track how accurately a student identifies brain regions, how efficiently they navigate a lesion mapping task, or how they respond to clinical decision points within a scenario. Makransky and Petersen (8) advocate for the use of learning analytics in immersive settings to generate actionable feedback and inform instructional design.

In addition to formative assessment, summative strategies should include reflective analysis. Encouraging students to articulate their clinical reasoning process following a VR simulation supports metacognitive development. These reflections can be scaffolded using tools such as concept mapping, narrative journaling, or debriefing sessions linked to program-level competencies. Furthermore, rubric-based evaluations aligned with Bloom's taxonomy and professional standards can provide consistent measurement of VR related outcomes. For instance, a rubric may assess a student's ability to synthesize neuroanatomical knowledge with occupational performance analysis in a given scenario. When used across modules, rubrics can also track longitudinal growth in clinical reasoning and decision-making.

Virtual reality can be effectively used to assess students' applied neuroscience knowledge through immersive Objective Structured Practical Examinations (OSPEs). In a VR-based OSPE, students interact with a 3D brain model and complete timed tasks that align with specific learning outcomes. These may include identifying neuroanatomical structures, explaining their functions, and linking them to clinical symptoms presented in a simulated case. This format encourages students to combine spatial understanding, knowledge recall, and clinical reasoning. Performance is evaluated using rubrics that measure accuracy, clarity of explanation, and application of theory to practice. VR-based OSPEs offer a structured, engaging, and repeatable alternative to traditional practical exams, supporting both content mastery and critical thinking (12, 13).

### Digital fluency and faculty preparedness

As emerging technologies redefine the landscape of rehabilitation education, there is a growing imperative to “level up” digital literacy across all stakeholders. Faculty must engage in ongoing professional development to design and facilitate immersive, technology-enhanced learning environments with pedagogical intention. Simultaneously, students must take active engagement of their learning by developing critical exploration and digital competencies necessary to navigate, critique, and apply these tools meaningfully (1).

In conclusion, when immersive learning is guided by clear, theory-informed learning outcomes and supported by appropriate assessment strategies, it can transform how neuroscience is taught in occupational therapy programs. Virtual reality allows for the integration of cognitive, affective, and psychomotor domains, preparing students not only to understand the brain but to think, feel, and act as clinicians. As immersive education continues to evolve, aligning learning objectives with assessment tools will be key to demonstrating its efficacy and sustainability in professional curricula (7).
